# Effect of Dexmedetomidine on Early Postoperative Cognitive Function in Patients Undergoing Arthroscopic Shoulder Surgery in Beach Chair Position: A Randomized Double-Blind Study

**DOI:** 10.3390/jcm11112970

**Published:** 2022-05-25

**Authors:** Namo Kim, Kwan Hyung Kim, Yong Seon Choi, Sei Han Song, Seung Ho Choi

**Affiliations:** 1 Department of Anesthesiology and Pain Medicine, Yonsei University College of Medicine, Seoul 03722, Korea; namo@yuhs.ac (N.K.); anekhkim@yuhs.ac (K.H.K.); yschoi@yuhs.ac (Y.S.C.); songseihan@yuhs.ac (S.H.S.); 2 Anesthesia and Pain Research Institute, Yonsei University College of Medicine, Seoul 03722, Korea

**Keywords:** arthroscopy, dexmedetomidine, inflammation, postoperative cognitive dysfunction, sitting position

## Abstract

This study sought to determine whether intraoperative dexmedetomidine infusion might reduce the incidence of postoperative cognitive dysfunction (POCD) and alleviate the neuroinflammatory response in patients who have undergone arthroscopic shoulder surgery. A total of 80 patients over 60 years of age who had undergone arthroscopic shoulder surgery in the beach chair position were randomly allocated to either the dexmedetomidine group (Group D) or the control group (Group C). Dexmedetomidine (0.6 μg/kg/h) or a comparable amount of normal saline was infused into each group during the surgery. The early incidence of POCD was assessed by comparing cognitive tests on the day before and 1 d after surgery. The neuroinflammatory response with the S100 calcium-binding protein B (S100β) assay was compared prior to anesthetic induction and 1 h following surgery. The incidence of POCD was comparable between groups D (*n* = 9, 22.5%) and C (*n* = 9, 23.7%) (*p* = 0.901). However, the results of the cognitive test revealed a significant difference between the groups after surgery (*p* = 0.004). Although the S100β levels measured at the end of surgery were significantly higher than those at baseline in both groups (*p* < 0.001), there was no difference between the groups after the surgery (*p* = 0.236). Our results suggest that intraoperative dexmedetomidine infusion neither reduce the incidence of early POCD nor alleviated the neuroinflammatory response in patients undergoing arthroscopic shoulder surgery.

## 1. Introduction

Postoperative cognitive dysfunction (POCD) is a syndrome characterized by a decline in performance on a neuropsychiatric test that significantly complicates the recovery of elderly patients following surgery, beginning days to weeks after surgery [1,2]. The incidence of POCD 7 days and 3 months following non-cardiac surgery was 26% and 10%, respectively, as shown by the International Study of Postoperative Cognitive Dysfunction (ISPOCD) [3,4]. It varies with clinical, demographic, and surgical variables, as well as the interval between surgery and assessment [1]. Since POCD can lead to poor functional recovery, prolonged hospitalization, long-term rehabilitation, and increased mortality [5], early detection and prevention of POCD in elderly patients are critical.

Dexmedetomidine is a highly selective α_2_ adrenoreceptor agonist that provides anxiolysis, sedation, and modest analgesia with minimal respiratory depression [6]. Dexmedetomidine has been demonstrated to be effective in ameliorating the incidence of delirium and POCD during sedation of intensive care patients [7]. However, the preventive effects of dexmedetomidine against POCD following surgery remain debatable [1,8] and there are no conclusive studies supporting the use of dexmedetomidine for the prevention of POCD.

Arthroscopic shoulder surgery is likely to result in hypoperfusion of the brain owing to the posture and hypotensive management requested by the surgeon. A previous study has suggested that unstable hemodynamics in elderly patients during epidural anesthesia for hip joint replacement surgery represents a risk factor for the development of POCD [9]. Along with the intentional hypotensive management for arthroscopic shoulder surgery, it can be hypothesized that there might be a causal neuronal damage with the change in neuroinflammatory markers such as gravitational effect, and that anesthesia-related hemodynamic changes can lead to cerebral hypoperfusion during the operation with implementation of the beach chair position (BCP) [10,11].

This study sought to investigate whether intraoperative dexmedetomidine infusion could reduce the incidence of POCD and alleviate the neuroinflammatory response in patients who underwent arthroscopic shoulder surgery.

## 2. Materials and Methods

### 2.1. Study Population

This study was approved by the Institutional Review Board of Severance Hospital, Yonsei University Health System, Seoul, Republic of Korea (No. 4-2015-0621) and registered at clinicaltrials.gov, accessed on 30 December 2015 (NCT02643017). After having obtained written informed consent, 80 patients scheduled for arthroscopic shoulder surgery under BCP were enrolled in this study, which was conducted between December 2015 and March 2018. Patients were randomly allocated to either the dexmedetomidine group (group D, *n* = 40) or the control group (group C, *n* = 40) via a computerized randomization table, and the surgeon and anesthesiologist were blinded to the patients’ group allocation.

Inclusion criteria were patients aged over 60 years of age who were scheduled for arthroscopic shoulder surgery with an American Society of Anesthesiologists physical status ranging from I to III, in addition to patients who could undergo a perioperative neurologic examination. Patients with severe pulmonary disease, heart failure (New York Heart Association class > II), atherosclerotic carotid stenosis, ischemic cerebrovascular disease, psychotic disorder, alcohol abuse, dementia, previous cognitive dysfunction, severe functional liver or kidney disease, uncontrolled diabetes mellitus, and a Korean version of the Mini-Mental State Examination (MMSE-K) score < 23 at the baseline examination were excluded from the study.

### 2.2. Anesthetic Management and Procedures

No premedication was administered to any of the patients. Standard monitoring, including pulse oximetry, electrocardiography, noninvasive blood pressure monitoring, and regional cerebral oxygen saturation (rSO_2_, INVOS 5100^TM^, Somanetics Corp., Troy, MI, USA), was performed prior to induction of anesthesia. An arterial catheter, used for continuous blood pressure monitoring and blood sampling, was inserted into the non-operating wrist. General anesthesia was induced using 1.0 to 1.5 mg/kg propofol, 0.5 to 1.0 µg/kg remifentanil, and 0.6 mg/kg rocuronium bromide, and anesthesia was maintained with 7.0 to 9.0 vol% desflurane inhalation and 0.01–0.2 µg/kg/min dose of remifentanil targeted at Bispectral index (BIS^TM^, Aspect Medical Systems, Norwood, MA, USA) between 40 and 60 after endotracheal intubation, respectively. The patients’ lungs were ventilated with 50% oxygen and the respiratory rate was controlled to target end-tidal carbon dioxide tension (EtCO_2_) at 35–40 mmHg.

After endotracheal intubation, the patient’s surgical position was changed to a beach chair position (BCP) in an approximate 70° head-up position. After the position was modified, radial artery pressure transducer was zeroed at the level of the ear tragus. For induced hypotension at the surgeon’s request, mean arterial pressure was adjusted to approximately 70% of the patient’s usual mean arterial pressure, not to fall below 60 mmHg, by increasing the dose of remifentanil administration. Patient’s usual mean arterial pressure was calculated as an average of three blood pressure measurements taken in the ward before surgery. If the mean arterial pressure decreased below 60 mmHg during the surgery, ephedrine or phenylephrine was administered.

The inhalation agent and remifentanil were discontinued 5 min prior to the completion of the surgery, and 1 μg/kg fentanyl was administered for pain management.

### 2.3. Study Design and Serum Biomarker Measurement

All of the enrolled patients were randomly assigned to receive either dexmedetomidine (Group D) or normal saline (Group C). In Group D, dexmedetomidine (Precedex™, Pfizer, New York, NY, USA) was diluted to a concentration of 4 μg/mL and administered intravenously at 0.6 μg/kg/h throughout the surgery. In Group C, a comparable amount of normal saline was administered. Hemodynamic parameters (mean arterial pressure and heart rate), including cerebral oxygen saturation, were recorded at the following time points: ① T0, before anesthetic induction; ② T1, 10 min after anesthetic induction; ③ T2, 60 min after changing to BCP; and ④ T3, 10 min after having returned to the supine position. The anesthesiologist was blinded to the study medications, which were administered after endotracheal intubation until completion of the surgery.

The S100 calcium-binding protein B (S100β) was used to assess neuroinflammatory responses. Arterial blood samples for S100β measurement were collected into EDTA tubes prior to anesthesia induction and 1 h after surgery in the post-anesthetic care unit. Blood samples were immediately centrifuged and stored at −80 °C for subsequent S100β measurements, which were carried out using an enzyme-linked immunosorbent assay (Human S100β ELISA, EZHS100 β-33K, Merck Millipore Corp., Billerica, MA, USA).

### 2.4. Neurologic Evaluation

Patients were screened and assessed on the day before and first day after surgery using the MMSE-K and a battery of visuomotor construction tests. All of the patients were assessed by the same anesthesia physician blinded to the use of dexmedetomidine and normal saline. The total score of the MMSE-K was 30 and the compositions were as follows: ① 10 scores of disorientation, ② 3 scores of memory, ③ 3 scores of learning, ④ 5 scores of mathematics or attention, ⑤ 6 scores of language, ⑥ 2 scores of executive function, and ⑦ 1 score of visual spatial skill. The battery of visuomotor construction tests (DLOTCA-G^TM^ battery, Maddak Inc., Wayne, NJ, USA) consisted of copying geometric forms, reproduction of a two-dimensional model, pegboard construction, colored block design, reproduction of a puzzle, and drawing a clock trial making test. Each test score was composed of a total of 30 out of 5 points, which were combined into a composite of cognitive outcomes.

Individual Z scores were computed for each test by subtracting the mean change score from the change score between baseline and follow-up for the corresponding test. This result was then divided by the standard deviation of the mean change score of each test to obtain the individual cognitive Z scores for each test. Composite Z scores were then assessed for each patient for the baseline and follow-up tests by averaging all Z scores from the MMSE-K and visuomotor construction tests. As such, a negative composite Z score reflects a decreased overall performance from baseline relative to the mean of all of the patients. Finally, postoperative cognitive dysfunction was defined as a composite z-score ≤ −1.96 [12].

### 2.5. Statistical Analysis

The primary outcome assessed was whether or not intraoperative dexmedetomidine infusion could reduce the incidence of POCD, and the secondary endpoint was whether dexmedetomidine infusion could alleviate the neuroinflammatory response. Based on the results of previous studies [13,14], the sample size was derived from the decrease in the incidence of POCD within a week from the null hypothesis of 60% to the alternative hypothesis of 30%, which corresponded to a total of 80 patients at a 2-sided α = 0.05, with 80% power.

The normality of the datasets was assessed using the Kolmogorov–Smirnov test. All data are expressed as means ± standard deviation, ranges, numbers and percentages, or medians and interquartile ranges. Data from the groups were compared using the chi-squared test, Mann–Whitney U-test, and Student’s t-test, as appropriate. Repeated variables were assessed using a linear mixed model with group, time, and the interaction between groups and time as a fixed effect and patient indicator as a random intercept. Post-hoc analysis with Bonferroni correction for within-group comparison versus baseline parameters and between-group comparison versus postoperative results was performed for multiple comparisons. Statistical significance was set to *p* < 0.05. Statistical analyses were performed using SPSS version 25 (SPSS Inc., Chicago, IL, USA).

## 3. Results

**Figure 1:**
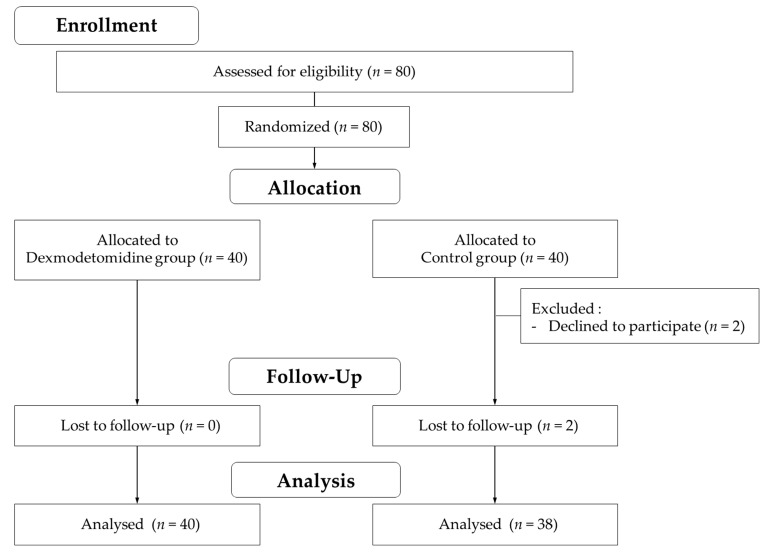
Patient enrollment flow.

A total of 78 patients were enrolled in this study (Figure 1).

The demographic characteristics were comparable in this study population (Table 1).

**Figure 2:**
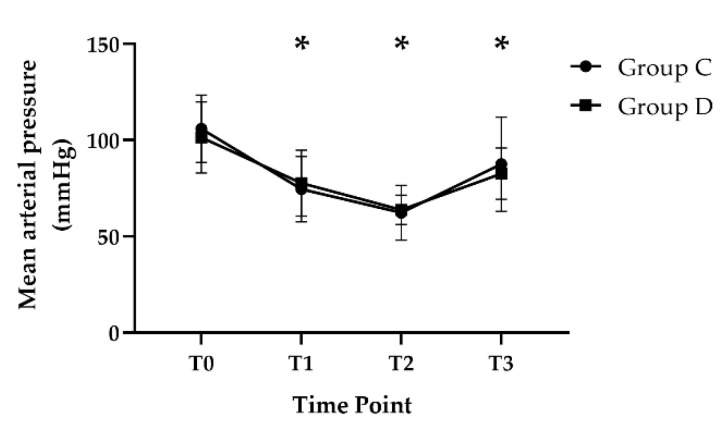
Change in mean arterial pressure during the study period. Error bars represent standard deviation. * *p* < 0.05 vs. T0. T0, before anesthetic induction; T1, 10 min after anesthetic induction; T2, 60 min after changing to beach chair position; and T3, 10 min after having returned to a supine position.

The mean arterial pressure at T1 (Group D, 77.7 ± 17.1; Group C, 74.6 ± 17.0), T2 (Group D, 63.8 ± 7.6; Group C, 62.3 ± 14.2), and T3 (Group D, 82.6 ± 13.3; Group C, 87.5 ± 24.4) was significantly lower than that at T0 (Group D, 101.4 ± 18.5; Group C, 105.9 ±17.5, *p* < 0.001) in both groups; however, there was no difference between the groups throughout the study period (Figure 2).

**Figure 3:**
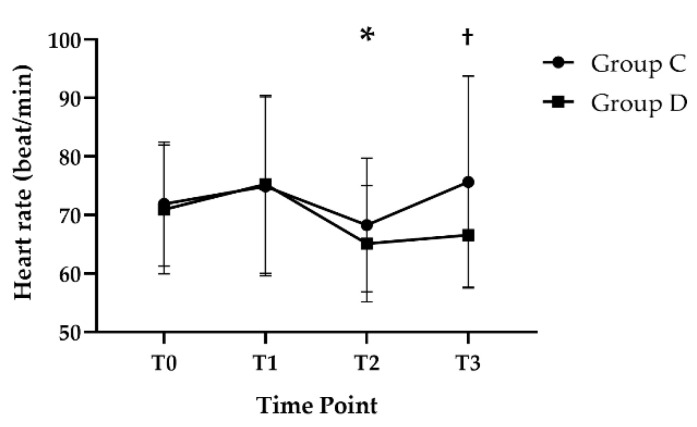
Change in heart rate during the study period. Error bars represent standard deviation. * *p* < 0.05 vs. T0, † *p* < 0.05 vs. Group C. T0, before anesthetic induction; T1, 10 min after anesthetic induction; T2, 60 min after changing to beach chair position; and T3, 10 min after having returned to a supine position.

The heart rate in Group D patients was significantly lower at T2 (65.1 ± 9.9) than at T0 (71.0 ± 11, *p* = 0.020) and showed a significant difference compared to those in group C at T3 (Group D, 66.6 ± 8.9; Group C, 75.7 ± 18.1, *p* = 0.021, Figure 3).

**Figure 4:**
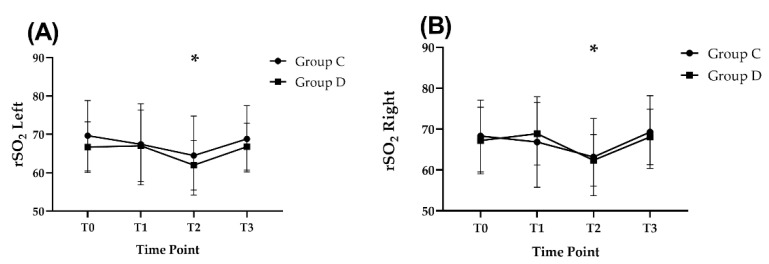
Change in regional oxygen saturation (rSO_2_) during the study period. (**A**) left rSO_2_, (**B**) right rSO_2_. Error bars represent standard deviation. * *p* < 0.05 vs. T0. T0, prior to anesthetic induction; T1, 10 min after anesthetic induction; T2, 60 min after changing to beach chair position; and T3, 10 min after having returned to a supine position.

The left and right rSO_2_ were significantly lower at T2 (left rSO_2_, Group D, 62.0 ± 6.5; Group C, 64.5 ± 10.3; right rSO_2_, Group D, 62.4 ± 6.3; Group C, 63.2 ± 9.5), than at T0 (left rSO_2_, Group D, 66.7 ± 6.6; Group C, 69.7 ± 9.2, *p* = 0.004; right rSO_2_, Group D, 67.2 ± 8.2; Group C, 68.3 ± 8.8, *p* = 0.006, respectively) in both groups, and were similar between the groups (Figure 4).

Among the 78 patients who completed the neurocognitive tests, 18 (23.1%) were diagnosed with POCD. The incidence of POCD was 9 patients (22.5%) in Group D compared to 9 patients (23.7%) in Group C, which was not significantly different (*p* = 0.901). The MMSE-K scores following surgery were comparable between the groups (*p* = 0.07). However, the scores of the visuomotor construction battery tests for those in Group D were significantly higher at the postoperative stage than for those in Group C (*p* = 0.004) (Table 2).

Although the results of S100β measured at the end of surgery (Group D, 62.5 [50.8–90.8] pg/mL; Group C, 69.3 ± 22.4 pg/mL) were significantly higher than at baseline (Group D, 32.9 ± 12.2 pg/mL; Group C, 36.8 ± 12.9 pg/mL; *p* < 0.001) in both groups, there was no significant inter-group difference following surgery (*p* = 0.236) (Table 3).

## 4. Discussion

In this prospective randomized double-blind study, intraoperative dexmedetomidine administration did not reduce the incidence of POCD and had no effect on alleviating the neuroinflammatory response induced by S100β. Cognitive deterioration began to appear on the first day after surgery.

A previous study reported that up to 60% of patients undergoing cardiac surgeries and 10% in major surgeries subsequently experience cognitive dysfunction months after surgery [10]. In addition to the several risk factors identified among patients [15,16], anesthesia remains one of the most important factors affecting the occurrence of POCD in patients undergoing surgery, regardless of the anesthetic technique that is used [17,18]. Furthermore, surgical stress results in both inflammation and immune activation, evidenced by localized reactions, as well as a systemic cascade of inflammatory signaling molecules with generalized inflammation. As perioperative neuroinflammation likely plays a pivotal role in the development of POCD, mitigating perioperative neuroinflammation with other potential factors, such as oxidative stress, may be of clinical importance to alleviate the development of POCD [19].

In addition to its use as a sedative, dexmedetomidine has been shown to have additional modulatory effects on neuroinflammation [20]. According to the previous studies, the mechanism by which dexmedetomidine attenuates neuroinflammation has been revealed into several categories [20]; First, dexmedetomidine inhibits the expression of inflammatory cytokines through the nuclear factor-κB pathway, which is a key regulator of the neuroinflammatory cascade and TNF-α activation [21]. Second, dexmedetomidine can reduce the hippocampal injury which seems to play a critical role in the pathogenesis of POCD, by enhancing upregulation of the anti-apoptosis protein along with downregulation of pro-apoptotic factors [22]. Finally, enhancement of the cholinergic anti-inflammatory pathway by dexmedetomidine can have a crucial role in modulation of neuroinflammation [23]. Preclinical studies in animal models have shown that dexmedetomidine provides neuroprotective effects and improves cognitive function after surgery [24,25]. However, clinical studies focused on preventing delirium or POCD by using dexmedetomidine remain controversial given the application of various inconsistent diagnostic criteria and neurologic endpoints [1,6,26,27]. As in previously published studies [1,27], the current trial did not show any preventive effect of dexmedetomidine in mitigating the incidence of POCD in patients undergoing arthroscopic shoulder surgery. No difference was identified between the two groups, which can be explained according to the following reasons. First, the median age of our study population was lower than that noted in previous studies. In a previous study by Deiner et al. [1], 12% of the study population experienced POCD, with a median age of 74 years compared with the average age of 65 years in our study population. Older age is one of the main patient-related risk factors for POCD. The effect may not have been seen in our study group given its overall younger age. Although we enrolled patients over 60 years of age, per the previous study [3,4], it can be assumed that the results would have differed from the current trial if we had enrolled patients over 70 years of age, since an advanced age is an independent risk factor for POCD. Second, the amount of dexmedetomidine infused might differ from that in previous studies. In the previous study by Su et al. [6], dexmedetomidine was administered as a low-dose infusion (0.1 μg/kg/h) without a loading dose. However, it was infused from the day of the surgery to postoperative day 1. Finally, the negative finding may have resulted from the relatively short intervals between the baseline examination and the postoperative examination of the MMSE-K and cognitive battery of tests. The POCD test is usually performed within seven days or before/at discharge from the hospital to abate the acute effects of hospitalization, anesthesia, and surgery [28]. However, in a previous study by Silbert et al., the authors reported that 30% of patients had neuropsychological changes within 18 h after cardiac surgery [29]. Based on the previous study showing that POCD-related information can be obtained earlier, our study performed a POCD test on postoperative day 1. As a result, the cognitive battery of tests demonstrated a significant difference between Groups D and C on postoperative day 1, which can be assumed to have begun to appear earlier than what was observed in previous reports [8]. These differences in test results did not lead to differences in the incidence of POCD clinically, although dexmedetomidine had a partial effect in alleviating this deterioration.

Compared with other inflammatory markers, S100β has been shown to be a POCD-related pro-inflammatory marker with interleukin-6 after cardiac and non-cardiac surgery [30]; in addition, it has been reported to be increased in patients with cognitive dysfunction after cardiac surgery [31,32]. Laflam et al. reported that there were no differences in S100β levels between baseline and postoperative values in either the lateral decubitus position or BCP in patients who had undergone arthroscopic shoulder surgery [33]. Notably, dexmedetomidine infusion did not alleviate the increase in S100β, since the magnitude of the increase in the level of the biomarker was comparable across groups. Nevertheless, our study revealed that serum S100β levels significantly increased after surgery in both groups. These results are consistent with the results obtained by Alatas et al. [34], which demonstrated the consequence of neuroinflammatory response after surgery which might be associated with brain injury. As the values of rSO_2_ at 60 min after changing to BCP were significantly lower than baseline in both groups, it can be assumed that it potentially caused hypoperfusion of the brain along with hypotensive blood pressure management, resulting in an increase in S100β levels.

Our study has several limitations. First, although we assessed the incidence of POCD using the MMSE-K test on the first day after surgery, more serial comparisons may have been needed for a certain period of time after surgery to probe whether the differences in the cognitive test between Group D and Group C showed clinical relevance. Second, the learning effect of both groups might have been underestimated given that there was no control group to compare that could adjust for these effects [35]. Third, although the current trial compared the S100β value between the groups rather than measuring the absolute value of S100β, the value of S100β might have been interfered by the shoulder surgery since it is also released from extracranial tissue [36]. Finally, as the current trial did not probe the patients’ own risk factors, future research will need to elucidate the relationships between potential risk factors and POCD incidence.

## 5. Conclusions

In conclusion, intraoperative dexmedetomidine administration did not reduce the incidence of POCD and had no effect on neuroinflammatory responses reflected in S100β levels. However, physicians should pay more attention to the development of POCD, since cognitive deterioration begins to appear by the first day after surgery.

## Figures and Tables

**Table 1:** Demographic and Characteristics of the Study Patients.

Characteristics	Group D (*n* = 40)	Group C (*n* = 38)	*p*-Value
Age (years)	65.0 ± 5.9	66.3 ± 6.3	0.344
Male/female	23/17	19/21	0.370
Height (cm)	162.1 ± 8.1	159.0 ± 8.1	0.089
Weight (kg)	63.7 ± 9.8	66.5 ± 11.5	0.249
Body mass index (kg/m^2^)	25.2 ± 3.1	25.1 ± 3.0	0.947
ASA classification 1/2/3, *n* (%)			0.471
1	5 (12.5%)	4 (10.5%)	
2	25 (62.5%)	21 (55.3%)	
3	10 (25%)	13 (34.2%)	
Hypertension, *n* (%)	17 (42.5%)	21 (55.3%)	0.373
Diabetes, *n* (%)	5 (12.5%)	7 (18.4%)	1.000
Intraoperative data			
Anesthesia time (min)	139.1 ± 31.6	133.0 ± 36.7	0.718
Operation time (min)	92.6 ± 31.3	90.0 ± 34.7	0.423
BCP duration (min)	104.5 ± 31.8	96.9 ± 29.0	0.281
Hypotensive duration (min)	73.3 ± 24.3	74.4 ± 27.9	0.857
Total remifentanil infusion (μg)	618 (363, 800)	622 (400, 700)	0.669
Total dexmedetomidine infusion (μg)	93.4 ± 26.0	-	<0.001
Total ephedrine dose (mg)	12 (8, 20)	12 (6, 18)	0.353
Total phenylephrine dose (μg)	0 (0, 37.5)	0 (0, 0)	0.685

Data were presented as mean ± standard deviation, numbers (percentages) or median (interquartile range). ASA = American Society of Anesthesiologists; BCP = beach chair position.

**Table 2:** The incidence of POCD and the results of neurologic and outcome.

Variable	Group D (*n* = 40)	Group C (*n* = 38)	*p* Value
POCD, *n* (%)	9 (22.5%)	9 (23.7%)	0.901
MMSE-K score			0.070
Pre-induction	28.0 (27.0–29.0)	28.0 (26.0–29.0)	
Post-operation	29.0 (28.0–29.0)	28.0 (26.0–29.0)	
Visuomotor construction battery test score			0.004
Pre-induction	55.0 (53.0–57.0)	55.5 (52.3–57.0)	
Post-operation	56.0 (53.3–58.0) *†	54.0 (50.0–56.0)	

Data were presented as numbers (percentages) or median (interquartile range). *p* group × time, linear mixed model analysis as a random effect and group, time, and group-by-time as fixed effects, * *p* < 0.05 vs. pre-induction, † *p* < 0.05 vs. Group C. POCD, postoperative cognitive dysfunction; MMSE-K, Korean version of Mini-Mental State Examination.

**Table 3:** Effects of dexmedetomidine on manifestation of S100β.

Variables	Group D (*n* = 40)	Group C (*n* = 38)	*p* Value
S100β (pg/mL)			0.236
Pre-induction	32.9 ± 12.2	36.8 ± 12.9	
Post-operation	62.5 (50.8–90.8) *	69.3 ± 22.4 *	

Data were presented as mean ± standard deviation or median (interquartile range). *p* group × time, linear mixed model analysis as a random effect and group, time, and group-by-time as fixed effects, * *p* < 0.05 vs. Pre-induction.

## Data Availability

Data are available from the corresponding author upon reasonable requests.
